# Rupture of sinus of Valsalva in a young laborer: A rare case report

**DOI:** 10.1002/ccr3.9184

**Published:** 2024-07-21

**Authors:** Muhammad Fawad Tahir, Abdul Munim, Sanila Mughal, Mah I Kan Changez, Munaum Ali Khan, Anum Yousaf, Shahzed Zakir

**Affiliations:** ^1^ Department of Medicine and Surgery HBS Medical and Dental College Islamabad Pakistan; ^2^ Department of Cardiology Rawalpindi Institute of Cardiology Rawalpindi Pakistan; ^3^ Department of Medicine Dow University of Health Sciences Karachi Pakistan; ^4^ Department of Surgery Quetta Institute of Medical Sciences Quetta Pakistan; ^5^ Department of Radiology Rawalpindi Institute of Cardiology Rawalpindi Pakistan; ^6^ Department of Medicine, Faculty of Medicine Nangarhar University Jalalabad Afghanistan

**Keywords:** physical exertion, right coronary sinus, rupture sinus of Valsalva, young adults

## Abstract

Ruptured sinus of Valsalva (RSOV) upon physical exertion is rare but should be considered in differential in young adults. Symptoms of acute heart failure, hemodynamic instability, and continuous heart murmur raises suspicion for RSOV and requires emergent surgical repair of right ventricular outflow tract.

## Graphical abstract

Transthoracic echocardiography showing a ruptured sinus of Valsalva of the right coronary cusp leading to the Left to Right shunt.

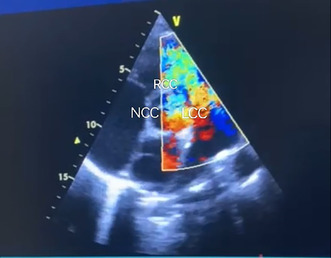

## 1 INTRODUCTION

Ruptured sinus of Valsalva (RSOV) is a rare cardiac anomaly accounting for 0.14%–3.5% of all open heart procedures.^1^ It can stem from either congenital abnormality or be acquired due to underlying connective tissue disorders. It is mostly associated with ventricular septal defect (VSD) or aortic regurgitation (AR). Symptoms appear once the coronary cusp ruptures into the cardiac chamber.^2^ Surgery is the mainstay for the treatment of patients with a RSOV. Herein, we report a rare case of RSOV in a 26‐year‐old male laborer, triggered by intense physical activity. To the best of our knowledge, there is a lack of reported cases in the literature about the RSOV due to intense physical activity.

## 2 HISTORY OF PRESENTATION

A 26‐year‐old male laborer presented to the emergency department with complaints of sudden onset of severe chest pain, shortness of breath, and dizziness. The onset of symptoms occurred 3 months before admission, initiating with a cough and progressing to severe dyspnea, for which he was referred to our hospital for further evaluation and management. However, he had a history of heavy weight lifting and strenuous physical activity at work. The patient experienced the onset of symptoms immediately following strenuous physical activity specifically through carrying heavy loads, thereby indicating it as a potential trigger.

On physical examination, the patient weighed 50 kg, height of 157 cm, and BMI was 21. The patient was pale and diaphoretic. The body temperature was recorded to be 98°F. He was tachycardic with a heart rate of 120 beats per minute, blood pressure was 148/100 mmHg, and a respiratory rate of 30 breaths per minute (bpm) with transcutaneous oxygen saturation (SpO_2_) of 99% in room air. A cardiovascular examination revealed a harsh pan systolic murmur heard best at the right upper sternal border. Lung sounds were clear, and the rest of the physical examination was unremarkable. The patient denied of a positive family history of any cardiovascular, systemic, congenital or any hereditary disorder.

## 3 PAST MEDICAL HISTORY

The patient reported no significant medical history and no history of congenital heart disease. He denied any past episodes of chest pain or cardiac symptoms.

## 4 DIFFERENTIAL DIAGNOSIS

Based on the clinical presentation, a differential diagnosis of VSD, atrial septal defect (ASD), aortic stenosis, and RSOV was made. These differential diagnoses were made based on the physical and clinical presentation of the patient. In order to identify the cause of RSOV, the patient was evaluated for all the acquired causative factors of RSOV. Based upon the absence of any clinical finding, that is, rash, skin lesions (chancre), mucous membrane involvement, and absence of syphilis‐associated risk factors, syphilis was eventually excluded as a trigger for RSOV. Similarly, the absence of cardiovascular risk factors such as hyperlipidemia, hypertension, diabetes, and smoking history, along with no identifiable plaque formation, calcification or atherosclerotic changes eventually led to the exclusion of arteriosclerosis. No remarkable findings on clinical examination and imaging studies also led to the exclusion of endocarditis and patent foramen ovale. Therefore, the RSOV was considered to be due to an acquired defect following heavy physical activity. The relevant laboratory findings are given in Tables 1, 2, 3, 4, 5, 6.

**TABLE 1:** Coagulation profile.

Parameter	Result	Reference range
PT	19.8	(10–14) s
INR	1.6	(0.9–1.3) ratio
APTT	26.4	(26–40) s

**TABLE 2:** Renal function test.

Parameter	Result	Reference range
Urea	71.00	(15–45) mg/dL
Creatinine	0.79	(0.2–1.1) mg/dL
Uric acid	9.3	(3.4–7.2) mg/dL
BUN	33	(8–22) m

**TABLE 3:** Lipid profile.

Parameter	Result	Reference range (mg/dL)
Cholesterol	101	<200
Triglyceride	187	<200
High‐density lipoprotein (HDL)	48	40–65

## 5 INVESTIGATIONS

The patient underwent a series of diagnostic tests, including an X‐ray chest (PA view), transthoracic echocardiography (TTE), and computed tomography angiography (CTA).

**FIGURE 1:**
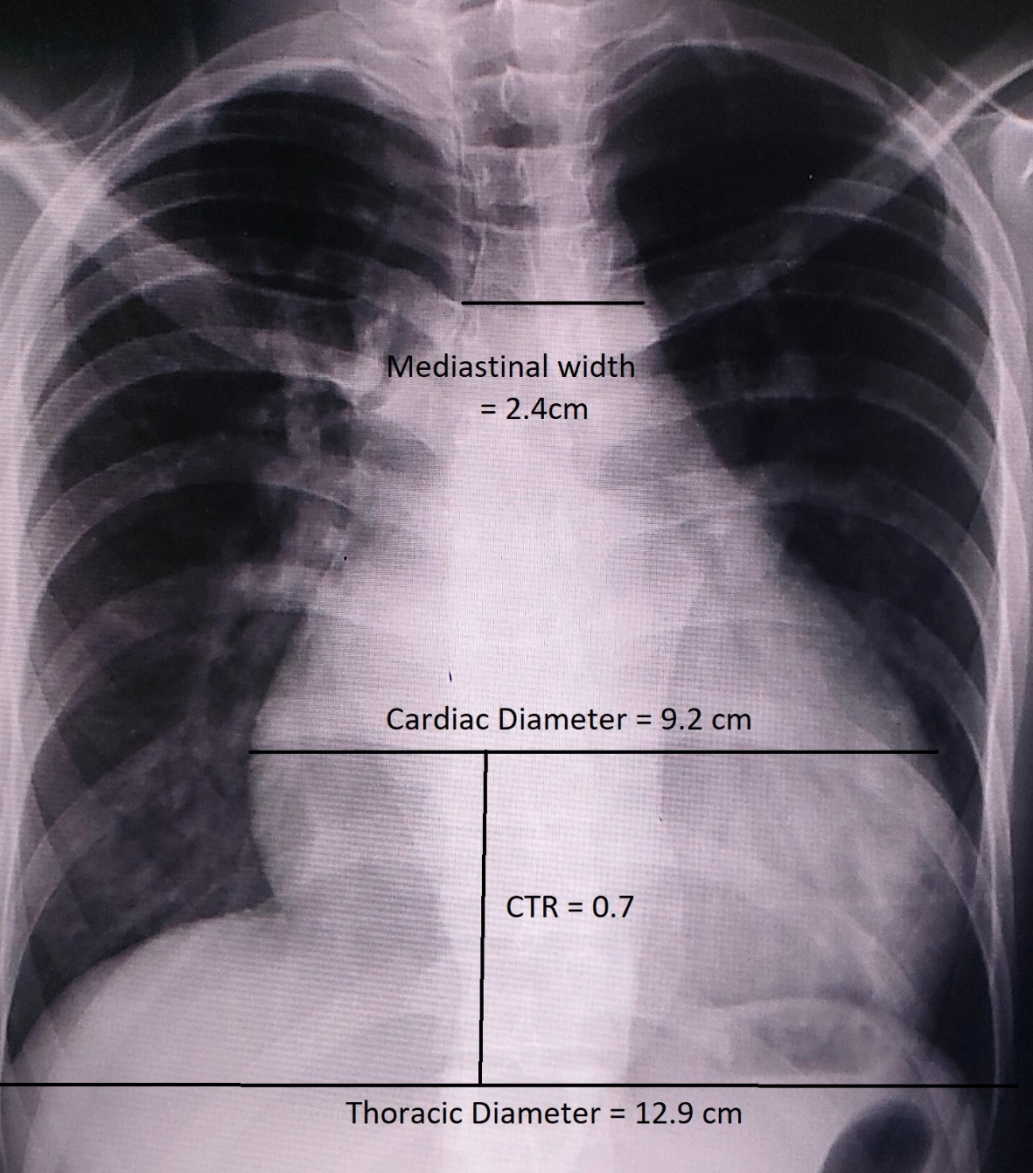
X‐ray chest (PA view). Posteroinferior view (PA view) of X‐ray chest showing obvious cardiomegaly as represented by the arrows with increased cardiothoracic ratio.

**FIGURE 2:**
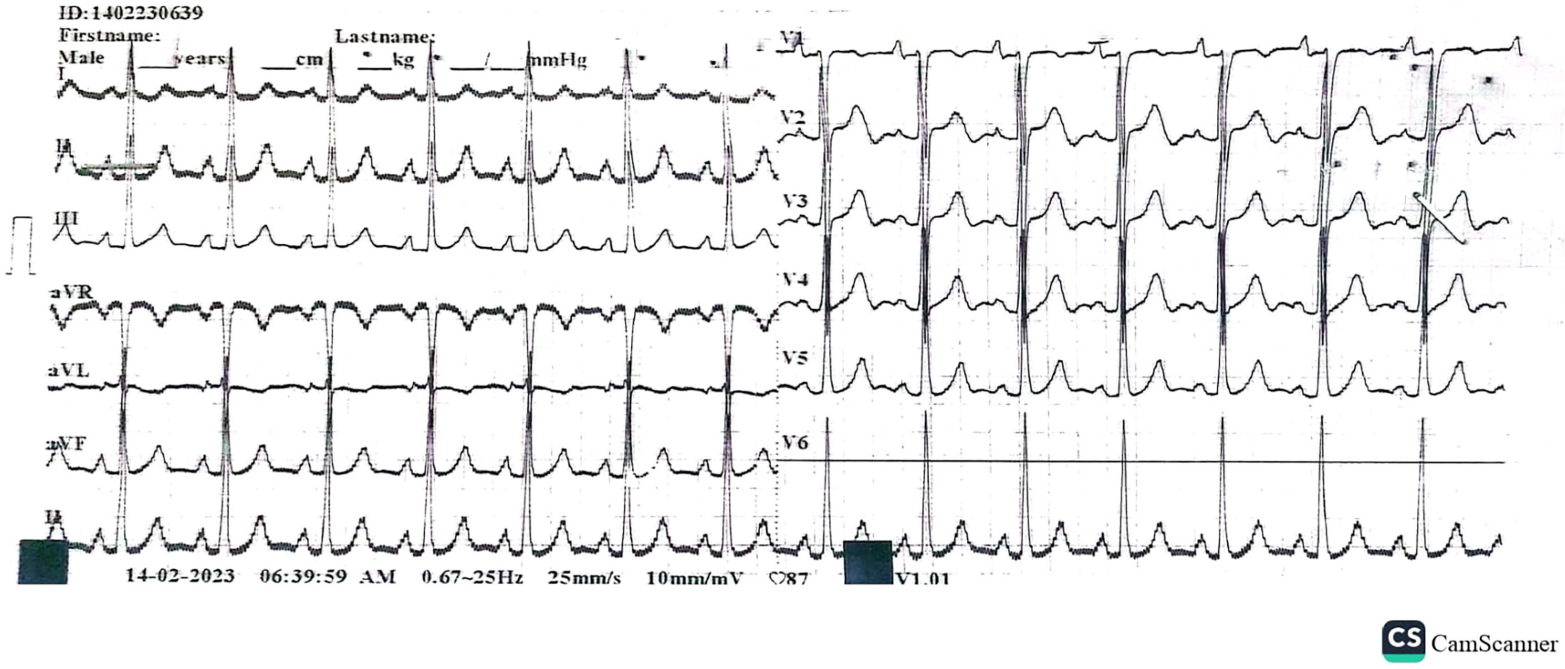
Preoperative 12 lead EKG.

**FIGURE 3:**
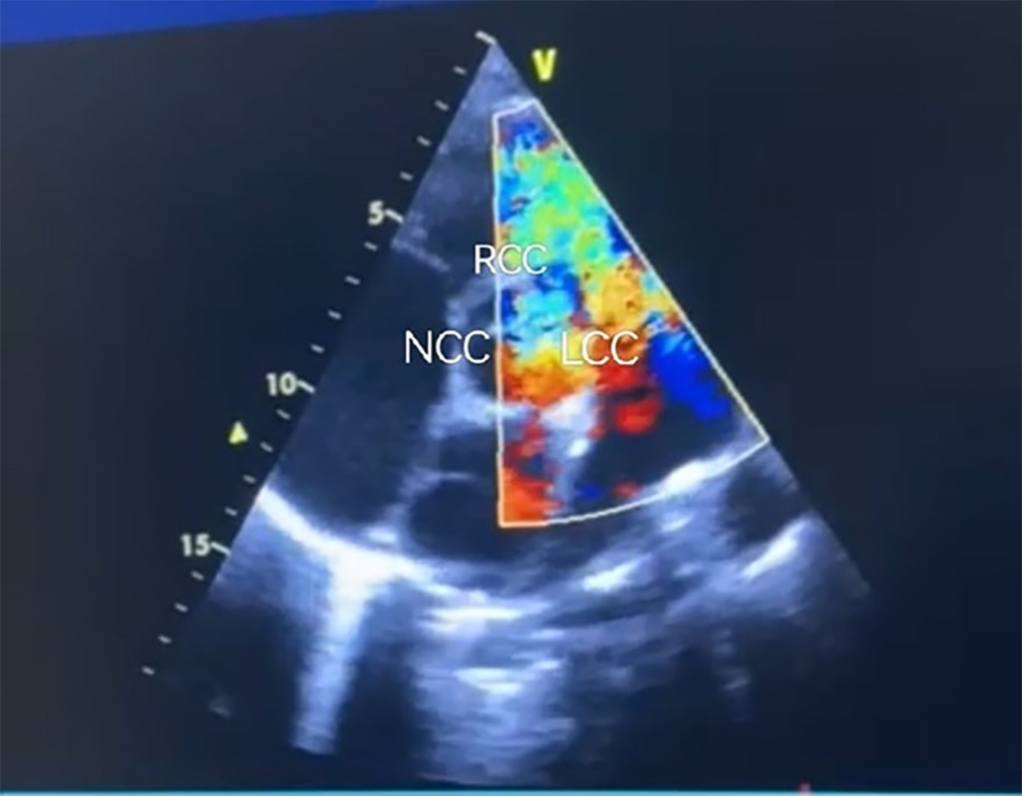
Transthoracic echocardiography. The arrow shows a ruptured sinus of Valsalva of the right coronary cusp leading to the left to right shunt.

An electrocardiogram showed sinus tachycardia with no evidence of acute ischemic changes. Chest X‐ray revealed prominent cardiomegaly with an increased cardiothoracic ratio (CTR) as shown in Figures 1 and 2. Laboratory investigations including complete blood count, basic metabolic panel, and cardiac enzymes were within normal limits. In addition, a TTE was performed, which showed left ventricular ejection fraction of 55%, right ventricular systolic function was normal with tricuspid annular plane systolic excursion of 17 mm, there was mild AR, severe tricuspid regurgitation (TR) with moderate pulmonary hypertension and pulmonary artery systolic pressure of 45 mm Hg. Other valves appear normal in structure and function, there was no clot and no pericardial effusion seen. Based on the clinical presentation and echocardiographic findings, a diagnosis of RSOV was made that was draining into the right ventricular outflow tract (RVOT) with a left‐to‐right shunt as shown in Figure 3.

**TABLE 4:** Serum electrolytes (Na, K, and Cl).

Parameter	Result	Reference range (mmol/L)
Sodium	135	136–145
Potassium	5.20	3.5–5.5
Chloride	104	98–110

**TABLE 5:** Cardiac enzyme profile.

Parameter	Result	Reference range (U/L)
CPK (creatine phosphokinase test)	66	0–190 38–174
CK‐MB	37.3	0–25
LDH	316	0–480
AST	49	0–43 0–48

**TABLE 6:** Complete blood profile.

Parameter	Result	Reference range
WBC	15.6	(4–10) 10^9^/L
RBC	4.81	(3.8–5.5) 10^12^/L
GRA	6.8	(1.6–7.6) 10^9^/L
LYM	6.8	(1.3–4.5) 10^9^/L
Eosinophils	0.1	(0.03–0.64) cells/μL
MON	1.8	(0.3–1) 10^9^/L
Basophils	0.1	(0–0.1) cells/μL
GR%	43.5	(43.6–75.4)%
LY%	43.9	(16.1–48.5)%
Eosinophils%	0.6	(0.6–7.3)%
MO%	11.6	(4.5–12.1)%
Basophils%	0.4	(0–1.7)%
HGB	14.3	(11.5–16.5) g/dL
HCT	44.4	(35–48.5)%
MCV	92.3	(84–98) fL
MCH	29.6	(27.5–32.4) pg
MCHC	32.1	(31.7–35) g/dL
Rdwc	14.4	(11.1–15)%
Rdws	45.9	(36.2–49.7) fL
PLT	281	(140–450) 10^3^/UL
MPV	8.4	(8.3–12.1) fL

**TABLE 7:** Aortic dimensions.

CT aortogram findings	Measurements
Aorta at the level of the aortic annulus	30 mm
Aortic root	33 mm
Ascending aorta	25.7 mm
Aortic arch	29 mm
Descending aorta	22 mm
Head and neck branches	Normal
Abdominal branches	Normal
Abdominal aorta	19.6 mm

**FIGURE 4:**
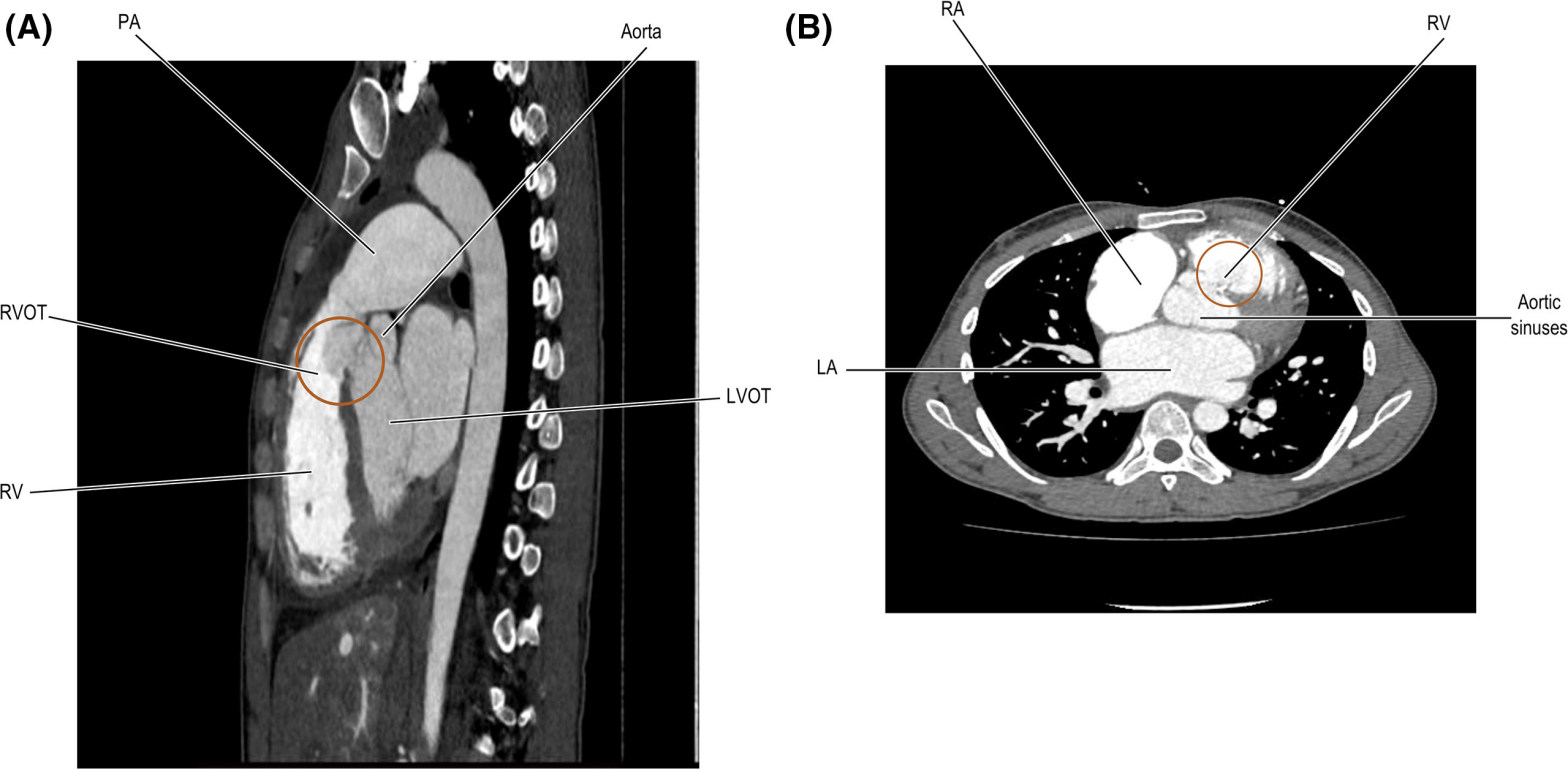
(A) CT‐scan imaging illustrating ruptured sinus of Valsalva (RSOV). (B) The CT scan reveals a fistulous communication resembling a long windsock between the right coronary cusp and the right ventricle, suggesting a RSOV. Additionally, signs of right heart strain were observed.

Moreover, a CT aortogram (CTA) was conducted, revealing a 1.8 cm defect in the right coronary cusp. The contrast entered the right ventricular (RV) cavity, confirming a rupture leading to an aorto‐RVOT fistula with a left‐to‐right shunt. In the RV cavity, there was a differential density contrast noticed. Both the right and left coronary arteries were adequately opacified, with the interventricular septum showing a slight flattening. The right atrium (RA) appeared to be of normal size. The pulmonary artery (PA) was found to be enlarged, measuring 3.8 cm, with a PA/aorta ratio of 1.3. Fluid density (18 HU) was observed in the anterior superior mediastinum. The cardiac size was enlarged, with a CTR of 162/220. Furthermore, there were minimal peri‐hepatic free fluids seen, and pulmonary congestive changes were evident. Table 7 lists the aortic dimensions measured through CTA. (Figure 4A,B).

## 6 MANAGEMENT

The surgical procedure involved median sternotomy, opening the pericardium, administering heparin, and placing aortic and bicaval cannulation for cardiopulmonary bypass (CPB) with hypothermia to 32°C. An aortic X clamp was applied, and retrograde cardioplegia was given to arrest the heart. The ruptured sinus was identified and examined, and no underlying VSD was found. The PA was opened, and the ruptured sinus draining into the RVOT was identified. A polytetrafluoroethylene (PTFE) patch was stitched to the aortic side of the sinus, and a Dacron patch was applied to the RVOT side of the sinus. The PA was closed, followed by the closure of the aorta. The rewarming and de‐airing of the heart were done and retrieved to normal sinus rhythm on mild inotropic support. Intraoperative TOE was done and there was no residual shunt across the repaired sinus patch. There was mild AR and the pulmonary valve was competent. The CPB was weaned off, protamine was given, decannulation was performed, and hemostasis was secured. A single RV pacing wire was placed, and two mediastinal drains were inserted. The chest was closed and the patient was shifted to ICU in stable condition. The patient's recovery after the surgery was smooth and without any complications. The patient was extubated within 5 h, and the drainage tubes were removed on the first day after the operation. The patient was able to move around well and was discharged from the hospital in excellent condition on the fifth day after the operation. Also, the wound on the chest appears to be healing well.

## 7 FOLLOW‐UP

After 1‐month follow‐up, the TTE revealed no residual shunt with mild to moderate AR and bicuspid aortic valve, the rest of the findings were normal. The chest pain was also resolved at follow‐up. The patient's current treatment plan was maintained and a subsequent follow‐up has been scheduled 1 month later to monitor any long‐term outcomes.

## 8 DISCUSSION

RSOV is a rare cardiac anomaly with an incidence of 1.4%–4.94% among Asians, most often presenting with aneurysmal dilated sinuses.^1^ It can either be congenital or acquired, with congenital cases being the most commonly reported.^2^ Congenital sinus of Valsalva is associated with connective tissue disorders; Marfan syndrome and Ehler–Danlos syndrome whereas the acquired cases can be due to myriads of underlying causes such as trauma, syphilis, hypertension, atherosclerosis, endocarditis, and cystic medial necrosis.^3^ In one instance, the right coronary sinus of Valsalva ruptured, causing left‐to‐right shunting and a breach into the RV outflow system. The fact that this rupture was accompanied by acute congestive cardiac failure, severe TR, mild AR, and moderate pulmonary hypertension emphasizes the complex and wide‐ranging effects of such an occurrence. The rupture into the RV outflow system was the cause of the rare combination of acute congestive heart failure, mild pulmonary hypertension, and severe TR in this instance. This case had a distinct set of clinical characteristics, even though right coronary cusp ruptures are known to be associated with a number of cardiac disorders, including ventricular septal abnormalities, aortic valve regurgitation, left ventricular outflow tract blockage, and more. Moreover, this patient was noticeably younger—26 years old at the time of presentation—despite the fact that the usual age range for sinus of Valsalva rupture is between 30 and 45 years old.^2^ In the case described above, the RSOV is considered acquired and believed to be due to intense physical activity, which is a rare presentation and, therefore, worth reporting and discussing. Up to date, there have been very few cases reported of rupture of the sinus of Valsalva due to intense physical activity or weight lifting.^4^, ^5^ In addition, the sinus of Valsalva most frequently originates in the right coronary cusp (70%–90%), followed by the noncoronary sinus (10%–25%) and, rarely, the left sinus (<5%)^2^ and the rupture usually occurs into the right ventricle, followed by the RA and rarely into the left ventricle, PA or interventricular septum.^2^, ^6^ Rupture of right coronary cusps and non‐coronary cusps has been observed to be associated with fistula formation and left‐to‐right shunting.^7^ As reported, rupture of right coronary cusps is most commonly associated with VSD in 50%–60% of the cases, aortic valve regurgitation (20%–30% of the cases), left ventricular outflow tract obstruction with subaortic membrane or bicuspid aortic valve (10%), pulmonary stenosis (5%), ASD (2%–5%), aortic coarctation (1%–2%); rarely tetralogy of Fallot, anomalous origin of a coronary artery, and patent ductus arteriosus, the unique features of this case contradicted these typical associations.^8^ This case is distinct not just because of the patient's advanced age but also because of the unusual clinical appearance brought on by physical activity. It is essential to promptly identify instances of this nature, as they frequently need a high degree of clinical suspicion. In the case described above, the patient was diagnosed with right coronary sinus of Valsalva rupture into the RVOT with left‐to‐right shunting and was identified to be associated with mild AR, severe TR, and moderate pulmonary hypertension along with acute congestive heart failure.

Diagnostic accuracy depends on the comprehensive anatomical insights provided by imaging modalities such as TEE or TEE with CTA. When there is a rupture, surgical intervention becomes essential since there is a median survival period of 1–2 years after the rupture, and delayed repair drastically lowers survival chances. The operative mortality is 1.9%–3.6% with 90% survival at 15 years after surgery.^9^ Furthermore, the unusual triggering event in this young patient deviates from the traditional correlation with preexisting aortopathies by happening after intense weight lifting. This aberration complicates the diagnostic process by raising the possibility of a connection between physically demanding activity and RSOV in otherwise healthy patients. Indications for surgery for unruptured SOVA are infective endocarditis, intractable arrhythmias, coronary artery compression, and outflow tract obstruction. The presence of left‐to‐right shunt, fistulization, and acute congestive heart failure, as was the case for our patient, are indications of emergent surgical intervention.^10^ A comprehensive differential diagnosis was required due to the diagnostic difficulties in this case, which originally included diseases such as VSD, ASD, aortic stenosis, and RSOV. The diagnosis of RSOV was finally guided by the unique characteristics seen, highlighting the need of meticulous clinical evaluation and examination of various clinical presentations in reaching an accurate diagnosis.

## 9 CONCLUSION

The RSOV due to intense physical activity is a rare finding but should still be taken into consideration in the differential in young and middle‐aged patients with symptoms of acute heart failure, hemodynamic instability, and a continuous heart murmur. This unique correlation highlights the need to investigate novel precipitants for cardiac events and broaden the diagnostic paradigm to guarantee timely and accurate interventions in such cases, ultimately contributing to our understanding of the diverse etiologies associated with cardiac complications.

## AUTHOR CONTRIBUTIONS

**Muhammad Fawad Tahir:** Conceptualization; data curation; writing – original draft. **Abdul Munim:** Conceptualization; data curation; writing – original draft. **Sanila Mughal:** Writing – original draft; writing – review and editing. **Mah I. Kan Changez:** Writing – original draft; writing – review and editing. **Munaum Ali Khan:** Supervision; writing – review and editing. **Anum Yousaf:** Supervision; writing – original draft. **Shahzed Zakir:** Supervision; writing – review and editing.

## FUNDING INFORMATION

None.

## CONFLICT OF INTEREST STATEMENT

The authors declare no conflicts of interest.

## CONSENT

Written informed consent was obtained from the patient to publish this report in accordance with the journal's patient consent policy.

## Data Availability

Data sharing not applicable to this article as no datasets were generated or analysed during the current study.
